# Is Fluorinating Polyethylene a Health Threat?

**DOI:** 10.1021/acscentsci.3c00498

**Published:** 2023-05-03

**Authors:** Britt E. Erickson

Kyla Bennett is fed up with the pollution flowing into her house through its water pipes. “I can’t drink my water. I’m not supposed to shower in my water. And we’ve been waiting now 2 years for this filtration plant to come on line. It’s costing us, the taxpayers, $9 million,” says Bennett, Director of Science Policy at Public Employees for Environmental Responsibility (PEER), an advocacy group.

**Figure:**
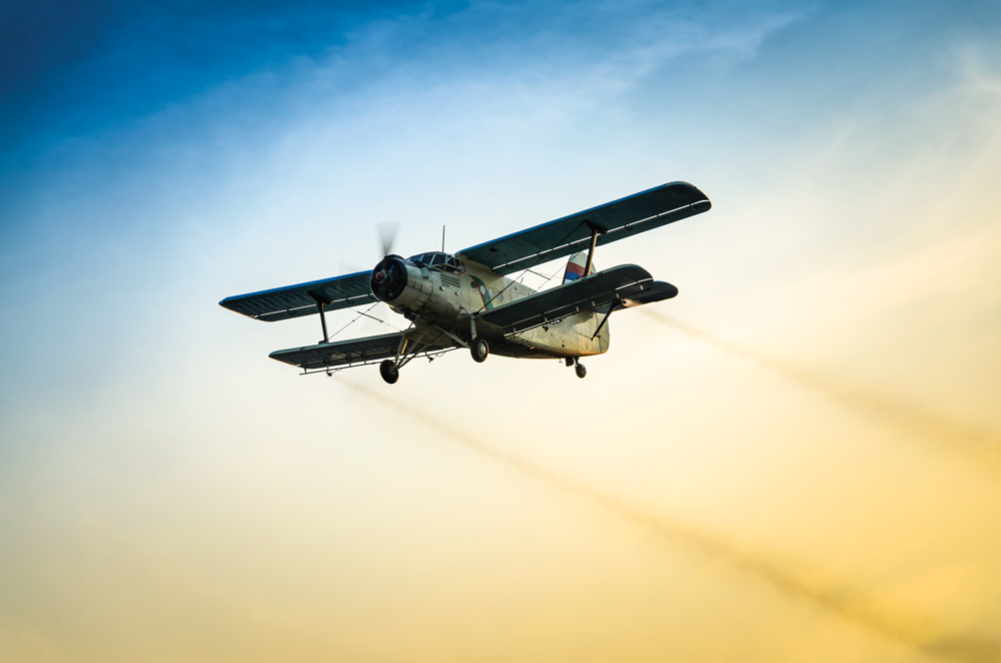
Pesticides sprayed to combat mosquitoes in Massachusetts were found to contain toxic fluorinated chemicals that leached from storage containers. Credit: Shutterstock.

Bennett lives in North Easton, Massachusetts, a small town just south of Boston. In 2019, she investigated drinking water contaminated with per- and polyfluoroalkyl substances (PFAS) from the use of firefighting foam on Fort Devens, a former military base northwest of Boston. She then tested her own water.

“I thought our water would be clean,” she says, noting that the town has no industry or firefighting training facilities. She also tested water from Sudbury, a town west of Boston next to a firefighter training site. “To my surprise, my water was more contaminated than Sudbury’s water,” she says.

Bennett suspected that a pesticide used to control mosquitoes was the source of PFAS in her water. She was right. “We’re at ground zero for triple E—eastern equine encephalitis,” a very rare but fatal mosquito-borne disease, Bennett says. “And we get sprayed continuously from planes with this pesticide called Anvil 10+10,” she says.

The Massachusetts Department of Environmental Protection and PEER identified PFAS in Anvil 10+10. The U.S. Environmental Protection Agency then confirmed in early 2021 that PFAS had migrated into the pesticide from the fluorinated high-density polyethylene (HDPE) containers it was stored in.

The EPA found some of the most toxic PFAS—perfluorooctanoic acid (PFOA) and other perfluoroalkyl carboxylic acids with chain lengths of eight carbons or more—in the pesticide. These long-chain PFAS are associated with developmental and immunological effects, certain cancers, and liver disease.

**Figure:**
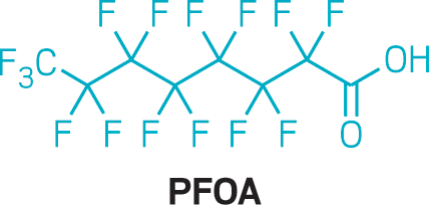
Credit: C&EN.

The agency concluded that the PFAS most likely formed during the fluorination process and that the containers were fluorinated after the plastic was molded. The primary company that performs such treatments is Inhance Technologies, a Houston-based firm that blasts plastic containers with fluorine gas in an oxygen-free chamber.

The process is much different than the more common method of producing fluoropolymers by polymerizing fluorinated monomers. That technology is also under scrutiny for producing PFAS.

Inhance says on its website that its Enkase barrier treatment “prevents permeation of contents through the packaging to the environment and preserves the quality of the product in the packaging.”

But manufacturing PFOA and certain other PFAS without notifying the EPA in advance violates a 2020 regulation under the Toxic Substances Control Act (TSCA), even if the PFAS are unintentional byproducts. The EPA is now going after Inhance for such violations. The agency filed a lawsuit in December, 2 years after it first approached the company for allegedly manufacturing PFAS.

PEER and the Center for Environmental Health (CEH) are also asking a court to stop Inhance from manufacturing PFAS and from distributing fluorinated plastic containers until the company complies with TSCA requirements.

In a statement, Inhance says that it “has never used or added PFAS as raw materials.” Testing results “suggested the potential for certain PFAS to be *unintentionally* produced in very low concentrations in the fluorination process as secondary reaction products—or impurities—that may remain with the HDPE containers,” the statement says (emphasis in the original).

The company says it has since adopted “process enhancements” to reduce PFAS concentrations to nondetectable levels. It is not providing details of the process, however, claiming that as confidential business information.

Environmental groups question Inhance’s claim, saying that the presence of any oxygen in its process can lead to the creation of PFAS. “We’re skeptical that Inhance has suddenly figured out how to get the oxygen out of there and not form PFAS,” Bennett says. Inhance argues that “the presence of oxygen or moisture during the fluorination process is not a potential source of PFAS in fluorinated barrier packaging.”

The environmental groups also say that contaminated pesticides are just the tip of the iceberg. Tens of millions of fluorinated HDPE containers have been filled and distributed to consumers and businesses, according to Bob Sussman, attorney for the CEH. The containers are marketed for storing many types of products, including agrochemicals, disinfectants, cleaning products, and food additives.

“Our goal in bringing this lawsuit is to stop the formation of PFAS during fluorination, which is a violation of the law, but even more than that is a significant public health issue,” Sussman says.

In January 2022, PEER filed a Freedom of Information Act request with the EPA, asking for data related to the formation of PFAS during fluorination of plastic containers by Inhance. The group also requested information on exposure to PFAS from the distribution and use of fluorinated plastic containers and on the health risks associated with PFAS from the fluorinated containers.

The EPA has yet to respond to the request, but it reported in September that PFAS can migrate from fluorinated HDPE containers into liquids stored in them, even water. The agency tested three brands of containers and found that PFAS levels varied with the brands, which it did not disclose. The EPA reported total PFAS of up to 15 ppb in methanol and up to 3 ppb in water. The concentration increased gradually with storage time.

Last year, the agency set an advisory limit of less than 1 part per trillion for PFOA in drinking water.

Inhance treats plastic containers with fluorine after the plastic is molded. But a few smaller companies produce fluorinated containers using an in-mold process, in which fluorine is added to the molten plastic before it is fully formed into a container.

It is unclear if PFAS are formed during these processes. “There needs to be more research into whether the in-mold fluorination leaches PFAS or not,” Bennett says.

One study, published in October, found that no detectable PFAS migrate from containers made using an advanced in-mold fluorination process. The study was funded by iPackChem, a French manufacturer of fluorinated plastic containers and other packaging products.

Environmental groups say the study is seriously flawed. “The methods do not even describe how the samples were selected or what advanced in-mold fluorination is,” says Tom Neltner, senior director of safer chemicals at the Environmental Defense Fund (EDF), an advocacy group.

**Figure:**
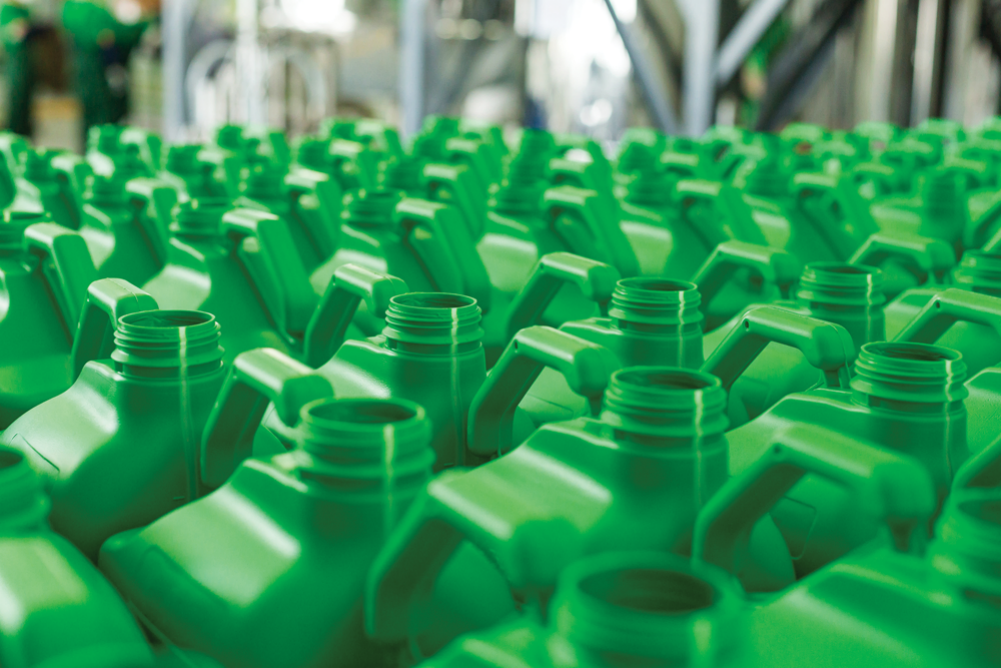
Processes for fluorinating the inside of high-density polyethylene containers are under scrutiny for creating toxic chemicals. Credit: Shutterstock.

The same month that the study was published, iPackChem acquired a majority stake in Kentucky-based TPG Plastics. In a press release, TPG said it will bring iPackChem’s advanced in-mold fluorination technology to North America, “with an initial focus on the crop protection market for the 2023–24 growing season.”

While the company’s initial focus is narrow, they may well expand into food, Neltner says. The EDF plans to file a petition with the U.S. Food and Drug Administration in the coming weeks to reverse approval of fluorinated polyethylene and polypropylene for food-contact use, he notes. The FDA granted the approval in 1983, before the health effects of low levels of PFAS were understood, he says.

The FDA warned manufacturers in August 2021 that only processes that keep out oxygen are allowed for fluorinating polyethylene containers used to store food.

Then, in July 2022, the FDA requested information on uses of fluorinated HDPE that involve contact with food. The agency received a letter from a law firm representing a major, though undisclosed, plastics fluorination company indicating that a small portion of fluorinated HDPE containers are used to store flavors and fragrances. FDA also received a letter noting that fluorinated HDPE is used to make gaskets and seals for milking machines.

In addition to the migration of PFAS from containers into food, recycling the containers could be a problem, Neltner says. “It raises questions because it’s still fluorinated HDPE. And it’s not labeled as such. It’s going to contaminate the recycling stream,” he says.

Inhance claims that Enkase leaves HDPE containers “fully recyclable.”

Fluorinated containers don’t raise concerns about the recycling of HDPE, say sources from the plastics recycling industry, including a physical chemist who asked not to be named to avoid being seen as representing the entire plastics recycling industry.

Fluorine gas treatment, like the one that Inhance provides, penetrates only a few nanometers deep into the HDPE. According to the sources, this leaves the plastic container with a maximum of a few parts per million of fluorine—not enough to interfere with the recycling process.

In addition, halogens can damage equipment used in pyrolysis- or other chemical-based plastics recycling methods, so recyclers often limit how much chlorine and fluorine they will accept. Buyers of mechanically recycled plastics—made by shredding and remolding used plastic—set similar limits.

Recyclers that use pyrolysis may also add calcium oxide to their process to convert unwanted halogens into calcium chloride and calcium fluoride. These salts become part of the char left after the pyrolysis process, the sources say.

Nevertheless, pyrolysis of fluorinated plastic could form toxic fluorine-containing compounds, says Neil Tangri, science and policy director of the Global Alliance for Incinerator Alternatives, which opposes pyrolysis-based plastics recycling.

If fluorinated HDPE containers are mechanically recycled, any PFAS they contain will remain in the material, says Judith Enck, president of the environmental group Beyond Plastics. If such recycled materials are mixed with virgin plastic, the end product is likely to be contaminated with PFAS, she says.

Environmental groups are frustrated that the federal government isn’t doing more to inform the public about PFAS in fluorinated plastic containers. “I don’t think FDA or EPA know the scope of the problem,” PEER’s Bennett says.

The CEH’s Sussman is concerned by the opacity of the matter. “EPA is not in a position to reveal any public information about the situation” because Inhance has very broad trade secret claims, he says. The EPA has had several years to review these claims but is essentially accepting them uncritically, he adds.

CEH’s top objective is to get the EPA “to come clean with the public about the nature of the problem and the extent of the health threat,” Sussman says.

## Additional reporting by Cheryl Hogue

.

*Britt Erickson is a senior correspondent at*Chemical & Engineering News*, the independent news outlet of the American Chemical Society. A version of this story appeared in C&EN.*

